# Esophageal ganglioneuromatosis; a rare cause of intractable esophageal stenosis: a case report

**DOI:** 10.1186/s12887-024-04923-8

**Published:** 2024-07-16

**Authors:** Mostafa Zain, Mohamed Abdelmalak, Saber Waheeb, Mohamed Mansy, Amir Ibrahim, Bassma El Sabaa

**Affiliations:** 1 Department of Pediatric Surgery, Faculty of Medicine, Alexandria University, Alexandria, Egypt; 2 Nile of Hope Hospital for congenital anomalies, Alexandria, Egypt; 3 Department of Pathology, Faculty of Medicine, Alexandria University, Alexandria, Egypt

**Keywords:** Ganglioneuromatosis, Esophageal stenosis, Dysphagia

## Abstract

**Background:**

Ganglioneuromatosis is a rare type of benign neurogenic tumor that usually affects the sites of the major sympathetic ganglia in the retroperitoneum and the posterior mediastinum. Affection of the gastrointestinal tract is rare, and involvement of the esophagus is exceptional. To the best of our knowledge, only 4 cases of esophageal ganglioneuromatosis in adults were reported in the literature. No cases have been reported in the pediatric age group.

**Case presentation:**

An 11-year-old boy presented with dysphagia due to severe esophageal stenosis caused by esophageal ganglioneuromatosis.

**Conclusions:**

Despite its rarity, the present case implies that ganglioneuromatosis should be considered in children with idiopathic esophageal stenosis.

## Background

Ganglioneuromatosis (GN) is a rare type of benign neurogenic tumor caused by extensive proliferation and hyperplasia of ganglion cells, Schwann cells, and nerve fibers [1]. It can be found anywhere in the body, especially at the sites of the major sympathetic ganglia in the retroperitoneum and the posterior mediastinum. Affection of the gastrointestinal tract is rare with the ileum, colon, and appendix being the commonly involved parts [2]. The clinical presentation is highly variable and depends on the site, size, extent of the lesion, and its effect on the motility of the intestine [3]. The involvement of the esophagus is exceptional. To our best of knowledge, only 4 cases of esophageal GN in adults were reported in the literature [4–7]. No cases have been reported in the pediatric age group.

In this article, we report a case of an 11-year-old boy with esophageal GN presented with dysphagia due to severe esophageal stenosis.

## Case presentation

An 11-year-old boy presented to our hospital with a long history of dysphagia. His condition started shortly after birth with repeated non-bilious vomiting. At the age of 2 years, the condition progressed to severe dysphagia. Investigation showed stricture in the middle esophagus and the boy has undergone several sessions of endoscopic esophageal dilatation outside our center.

**Fig. 1:**
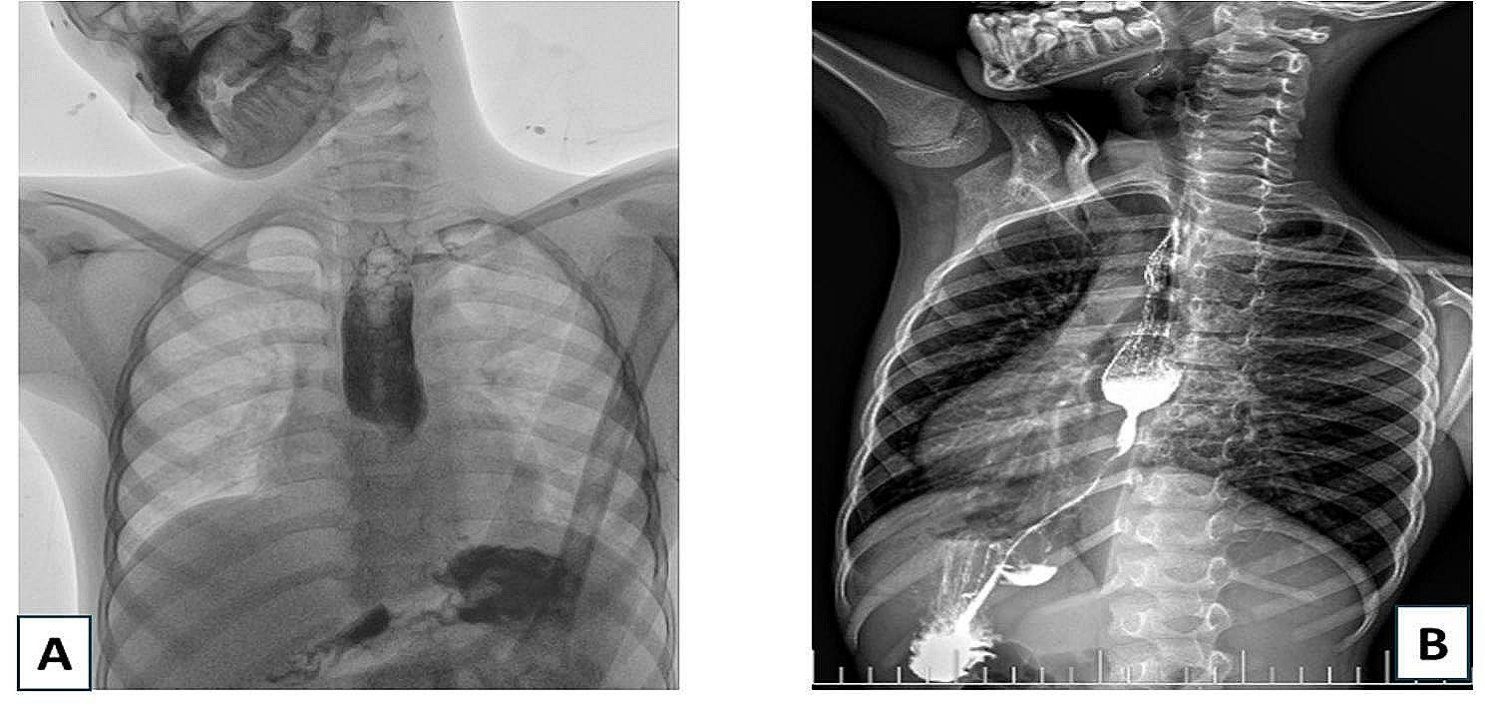
(**A**): Fluoroscopy. (**B**): Contrast swallow, oblique view. Both show severely stenosed central lumen at the lower third of the esophagus with proximal dilatation and shouldering

When presented to our hospital, the boy had severe dysphagia to solid and was dependent on fluids for his nutrition. His weight was only 21 kg which is much below the average for his age. A contrast swallow study and fluoroscopy were done and showed a severely stenosed central lumen at the lower third of the esophagus with proximal dilatation and shouldering (Fig. 1). Upper gastrointestinal (GI) endoscopy revealed non-dilatable pinpoint stricture at the distal esophagus with failure of the endoscope to pass through it. A computed tomography scan with contrast demonstrated diffuse circumferential thickening of the wall of a segment in the lower esophagus about 2 cm in length.

**Fig. 2:**
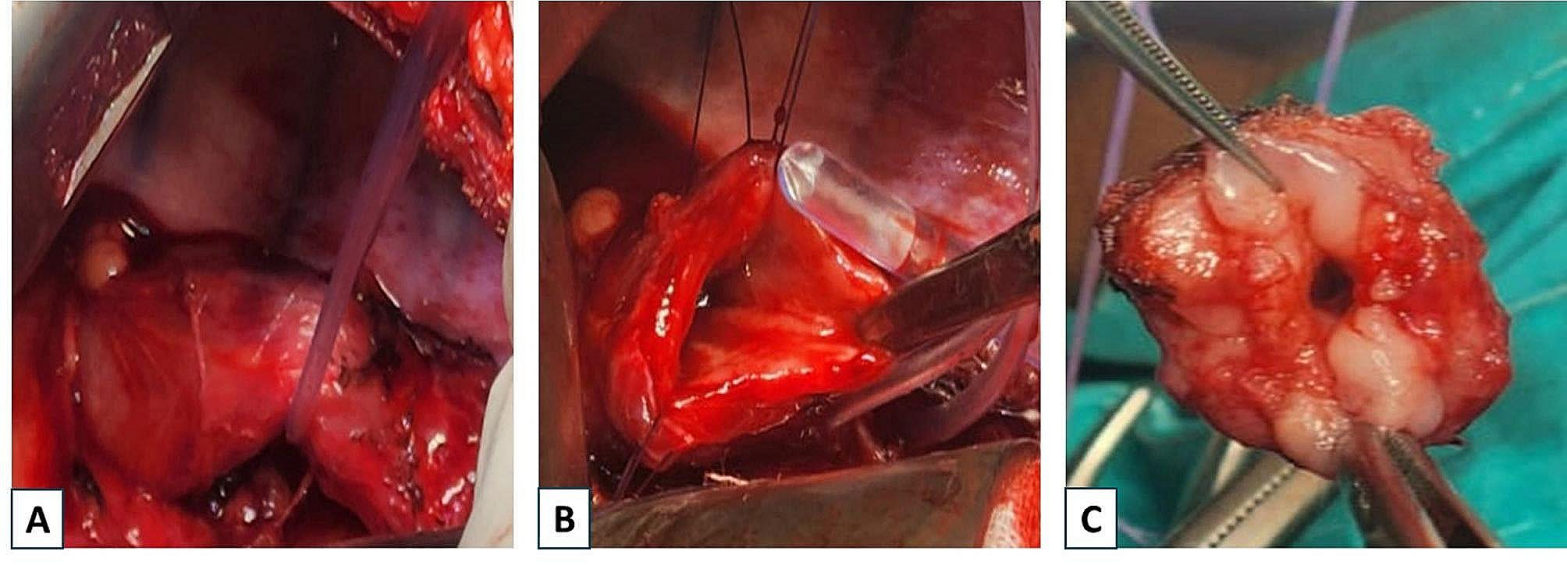
Intraoperative findings. (**A**): Thickened esophageal segment. (**B**): Upper segment with dilated lumen. (**C**): Lower segment with diffusely thickened wall with a central severely narrowed lumen

The initial diagnosis was thought to be a congenital stenosis or a tracheoesophageal remnant. The decision was made for surgical resection of the narrow segment. Through a right thoracotomy at the level of the 5th intercostal space, exploration revealed a segment with a thickened hard wall in the lower esophagus. The wall of the esophagus was opened above this segment. The distal segment showed a diffusely thickened wall with a central severely narrowed lumen while the upper segment showed a dilated lumen with a normal wall thickness (Fig. 2). The length of the narrow segment was about 1.5 to 2 cm. This narrow segment was resected, and an end-to-end anastomosis under mild tension was done with an overlying pedicled parietal pleural flap developed from the lateral side of the chest wall. A chest tube was inserted to drain any fluid collection in the chest. The patient showed an uneventful recovery.

**Fig. 3:**
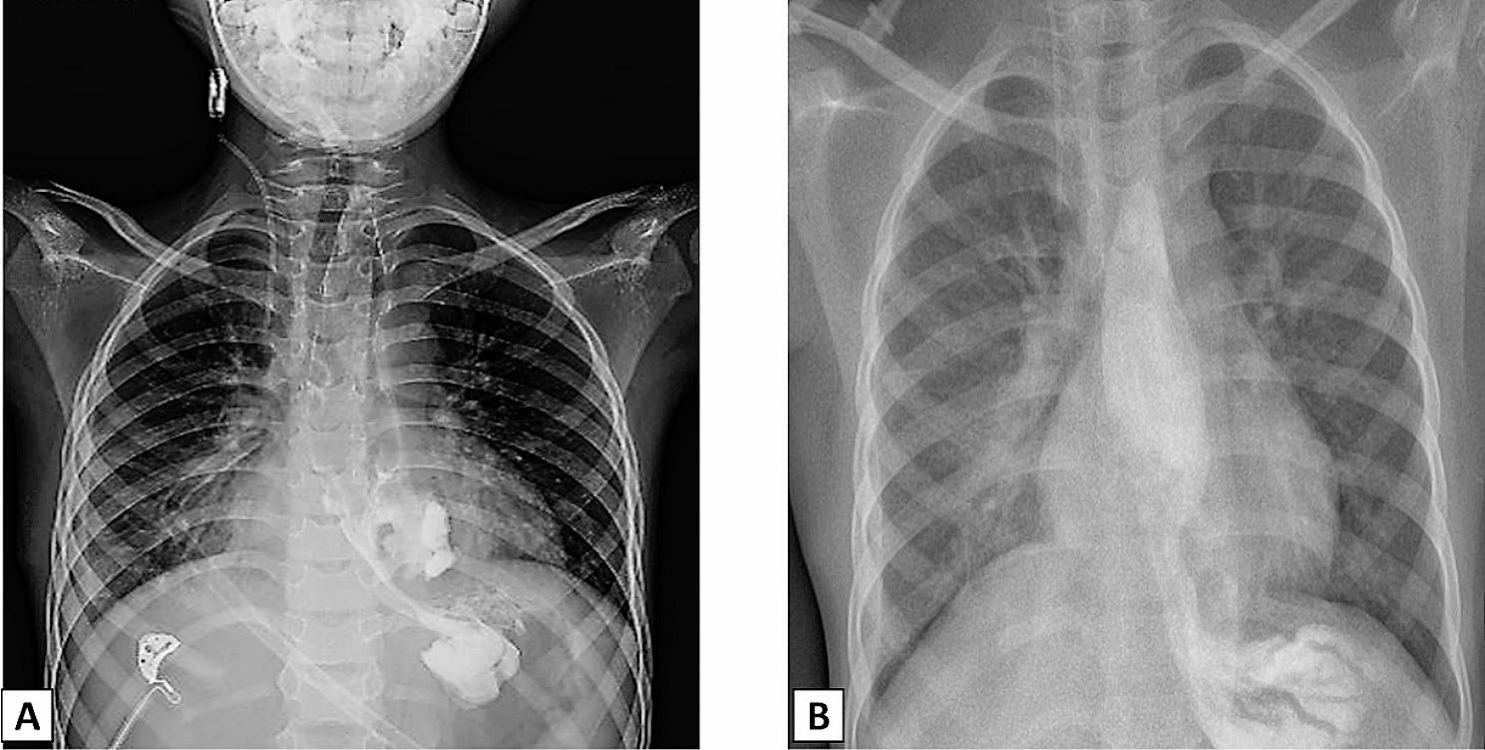
(**A**): Gastrograffin swallow done on the 5th postoperative day showing passage of the contrast to the stomach with leakage of the contrast into the mediastinum. (**B**): The study was repeated after one week and showed smooth passage of the contrast throughout the esophagus into the stomach without any leakage

After the operation, the boy was admitted to the Pediatric Intensive Care Unit (PICU). He was kept off oral with total parenteral nutrition (TPN). On the 5th postoperative day, an oral contrast study was done. It showed leakage of the contrast into the mediastinum (Fig. 3). So, starting oral feeding was deferred and TPN was continued. After another 7 days, the oral contrast was repeated and showed smooth passage of the contrast throughout the esophagus into the stomach without any leakage. Oral feeding was initiated and upgraded slowly. On the 15th postoperative day, the boy tolerated full oral feeding. The chest tube was removed on the 16th post-operative. The patient was discharged on the 18th postoperative day.

**Fig. 4:**
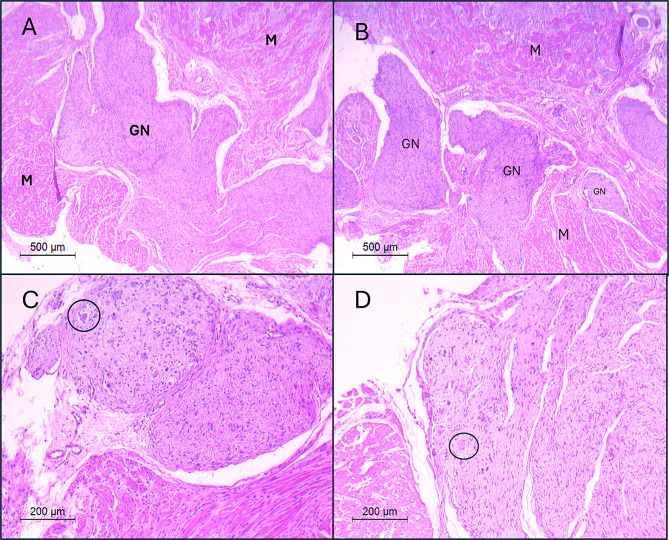
(**A**&**B**): “GN” Ganglioneuroma dissecting through bundles of muscularis propria “M”, H&E- X100. (**C**&**D**): Plump ganglion cells “black circle”, H&E-X200

Histopathological examination of the resected segment demonstrated; a transmural vague distribution of confluent mature ganglion cells and spindle cells with schwannian features (Fig. 4). Immunohistochemistry staining revealed a strong affinity of the ganglion cells for GFAP and S-100 while the spindle cells showed a strong affinity for S100 only. There was no evidence of ulceration or mitosis. It was classified as diffuse ganglioneuromatosis according to WHO 2019 classification.

After the diagnosis was made, the patient was evaluated to exclude other lesions or associated syndromes. General physical examinations, blood tests, fecal occult blood test, colonoscopy, and magnetic resonance enterography were negative. Also, genetic analysis for NF1, RET, and PTEN genes was done, and all gave negative results for any specific mutations. The patient was followed up regularly for a year. His weight increased by 5 kg in the first 6 months after the operation. He showed a smooth course without any complaints.

## Discussion and conclusions

GN of the gastrointestinal tract is a rare pathology featuring an abnormal proliferation of ganglion cells in the wall of the intestine with the surrounding Schwann cells and nerve fibers. It can affect any portion of the gastrointestinal tract, but the commonly affected parts are the colon, ileum, and appendix [8]. Affection of the esophagus is exceptionally rare.

Intestinal GN is classified according to WHO 2019 classification into three types. First, polypoid GN consists of sessile or pedunculated small polyps and is mostly seen in the colon. The mass is mucosal and may extend into the submucosa, but not transmural. This type is not associated with other syndromes and is considered hamartoma [9].

Second, ganglioneuromatous polyposis, which consists of numerous sessile or pedunculated mucosal and/or submucosal lesions. It is usually found in the colon and distal ileum. Its association with other systemic syndromes is unclear [10].

Third, diffuse GN is defined as disseminated, nodular, infiltrative intramural, or transmural proliferation of the neural elements in the wall of the ileum or the colon. It may be isolated or associated with syndromes. The transmural diffuse type is usually associated with genetic syndromes such as Neurofibromatosis 1, multiple endocrine neoplasia type 2B, and Cowden syndrome [10]. Reported cases with isolated GN are rare and not usually associated with other genetic disorders [9]. Our case was classified as diffuse transmural GN without having any signs of other syndromes or genetic disorders.

Congenital stenosis of the esophagus is a very rare malformation characterized by an intrinsic narrowing of the distal esophageal lumen due to the presence of tracheobronchial remnants, fibromuscular hypertrophy, or a membranous diaphragm. Although the stenosis is present at birth, the onset of symptoms is usually delayed till the age of 4–10 months with the introduction of solid food. The first line for treatment is endoscopic dilations of the stenotic segment or endoscopic resection of the web. Surgery is indicated after failure of conservative measures or in cases with tracheobronchial remnants [11]. In our case, the provisional diagnosis was congenital stenosis with a tracheobronchial remnant based on the findings of the imaging.

The clinical presentation of GN is highly variable as it depends on the type, site, size, and rate of growth of the lesion. It may be asymptomatic or may present with non-specific abdominal pain, obstruction, bleeding, or constipation [12].

Diagnosis of intestinal GN is extremely challenging as it is very rare, doesn’t have specific clinical signs or symptoms, and doesn’t show specific endoscopic morphological features. The histopathological examination is the only method that can provide a definitive diagnosis [9]. The differential diagnosis has a wide spectrum and depends on the clinical presentation. It includes Hirschsprung’s disease, inflammatory bowel disease, tuberculosis, tumors like adenocarcinoma, lymphoma, gastrointestinal stromal tumor, leiomyoma, and Cytomegalovirus infection [12].

Management of GN is not standardized as it is dependent upon the size, location, and accompanying symptoms of the intestinal GN. Some authors have documented that endoscopic complete resection proved to be a successful treatment for solitary GN [12]. In other reports, severely symptomatic cases required resection of the involved intestinal segment [10]. In our case, the decision was made for surgical resection of the stenosed esophageal segment after failure of multiple trials of endoscopic dilatation.

Management of leakage after esophageal anastomosis is highly variable with each center having its own protocol. Many factors are considered such as the primary pathology, intraoperative findings, localization, onset and size of the leakage, the severity of symptoms, and the general condition of the patient [13]. Recently, practice has progressively moved away from aggressive early surgery toward more conservative management. There is a general agreement that conservative treatment should be the first choice in cases with asymptomatic or minimally symptomatic leakage while surgical intervention should be considered in cases with early leakage and/or severe sepsis [13].

In conservative management, the patient is kept off oral with parenteral or enteral nutrition through a jejunostomy for 1–3 weeks [14]. It is critical to establish adequate mediastinal drainage if there is any fluid collection [15]. Systemic treatment usually consists of broad-spectrum antibiotic therapy according to infectious parameters. Some authors suggested the use of anti-acids, anticholinergic medication, and prokinetics to reduce the amount of saliva and leakage [16].

Due to the rarity of the condition, the literature data about GN are scarce, especially in the pediatric population. The risk of recurrence is still unknown yet can’t be excluded, making the ideal follow-up plan a matter of considerable debate concerning the type of investigation to perform and the time intervals to adopt. In the reported cases of intestinal GN, some authors followed their patients with esophagogastroduodenoscopy, colonoscopy, and MR-enterography while others suggested a more conservative plan as GNs are a benign condition with excellent prognosis [12, 17]. Shekitka et al. reported a follow-up study of 28 patients with solitary GNs for 8 years during which none of the patients developed recurrence, NF1 disease, MEN2B, or other complications [10].

To conclude, only a few cases of intestinal GN in the pediatric age have been reported in the literature, and to the best of our knowledge, this is the first reported case of pediatric esophageal GN. Despite its rarity, the present case implies that this pathology should be considered in children with idiopathic esophageal stenosis.

## Data Availability

The datasets used and/or analysed during the current study are available from the corresponding author on reasonable request.

## Abbreviations

GI: Gastrointestinal
GN: Ganglioneuromatosis
PICU: Pediatric Intensive Care Unit
TPN: Total parenteral nutrition
